# Editorial: Natural Antimicrobial Peptides: Hope for New Antibiotic Lead Molecules

**DOI:** 10.3389/fphar.2021.640938

**Published:** 2021-02-16

**Authors:** Shaikh Jamal Uddin, Jamil Ahmad Shilpi, Lutfun Nahar, Satyajit D. Sarker, Ulf Göransson

**Affiliations:** ^1^ Pharmacy Discipline, Life Science School, Khulna University, Khulna, Bangladesh; ^2^ Centre for Natural Products Discovery, School of Pharmacy and Biomolecular Sciences, Liverpool John Moores University, Liverpool, United Kingdom; ^3^ Division of Pharmacognosy, Uppsala University, Biomedical Center, Uppsala, Sweden; ^4^ Department of Medicinal Chemistry, Uppsala University, Biomedical Center, Uppsala, Sweden

**Keywords:** natural antimicrobial peptides, antimicrobial drug resistance, natural products, antibiotic, drug discovery

Infectious diseases are serious global health problems as they are often difficult to control, manage or eradicate. COVID-19 pandemic is a classic example of such problems that has devastated the whole world. Lack of new drugs as well as resistance to available anti-infective drugs, particularly antibiotics, have made these diseases difficult to treat (World Health Organization, 2020). In fact, antibiotic resistance is one of the prevalent threats to global health today. Therefore, discovery of effective and safe antibiotics has become a global priority.

Natural products have evidently played an essential role in providing medicinal care and new therapeutics. About 87% of all human diseases, including bacterial infections, parasitic infections and immune disorders have been treated by natural products (Amedei and Niccolai., 2014). Natural products and their extensive chemical diversity make them significant sources to provide novel compounds for future drug development.

Natural antimicrobial peptides (NAMPs) belong to different peptide or protein families that are found in all classes of life as a part of innate immune responses as well as barrier defense (Diamond et al., 2009). Antimicrobial peptides (AMPs) have been reported to act as broad spectrum antibiotics that kill bacteria, viruses, fungi as well as cancerous cells and demonstrated as a potential source of new antibiotics (Ageitos et al., 2017). Because of their promising activity and future prospects, over the last twenty years there has been growing interests in AMPs research and therefore the list of identified NAMPs has been growing day-by-day. Compared to conventional antibiotics, NAMPs have more unique features (Mahlapuu et al., 2016). Exceptional structural and functional properties of NAMPs may make them a substitute for conventional antibiotics. NAMPs have also been found to be stable in various conditions and possesses low to no toxicity in humans which make them prospective candidate to develop new antibacterial leads (Bondaryk et al., 2017). Natural sources including plants, microbes, fungi, archaea, protists, and animals are the primary origin of NAMPs. These natural resources could have ethnomedicinal uses against infections which open essential clues for the discovery of new NAMPs. Recent reports demonstrated that more than 1,500 NAMPs have been identified from nature and a number of these NAMPs currently under clinical or preclinical trials including kalata B1 and B2, novexatin, omiganan, pexiganan, thionins, and thioneinetc (Salas et al., 2015; Molchanova et al., 2017; Gründemann et al., 2019).

Plants are capable of producing structurally diverse of compounds which serves as a specific function for plant itself including their communication, protection, defense or growth. The protection of plant from pathogenic infections have already been reported that they can develop different constitutive and inducible protection mechanisms via morphological barriers, secondary metabolites or AMPs (Benko-Iseppon et al., 2010). From traditional knowledge human have learned to use these chemicals in their own needs especially as a form of medicines. These secondary metabolites of plants have been served as a treasured of bioactive constituents that used in traditional medicines for the treatment of infectious diseases since ancient time. It is reported that plants are an important source of different AMPs and a number of AMPs have already been identified from different parts of plant which discovered via using the knowledge of their ethno-medicinal or traditional uses against infection (Nawrot et al., 2014). This is how plants interacted with people in their ethno-medicinal uses against infection. Therefore, ethnobotanical research has a great significance in new drug discovery and development such as the discovery of artemisinin from *Artemisia spp* (Kayser, 2018). Ethnobotanical research can also offer natural chemical libraries with diverse structures.

Due to rapid raising of antibiotic resistance, new emerging tactics are compulsory to develop new antimicrobial drugs. Discovery of new antimicrobial agents through scientific evaluation of traditional medicines involved ethnopharmacological approaches (Cox and Balick, 1994). Ethnopharmacology has already proved to be an innovative approach for drug discovery (Süntar, 2020). The discovery and development of a number of modern drugs were the credit to ethnopharmacology. Ethnopharmacological approaches focus on the people-nature interface and covers the past, present and future uses of natural resources that has contribution in maintenance of human health by opening essential clues for new drug discovery. The ultimate goal of this research topic is to update knowledge on NAMPs and their ethnomedicinal uses in preventing and managing infections in human, plant or animal, and future research directions for the discovery of new antibiotics from NAMPs. This special issue consists of six articles that cover diverse areas of research related to NAMPs and ethnopharmacology. A number of review articles in this issue have focused the reports on the correlation of ethnobotanical/ethnopharmacological data with the exploration of new AMPs. It also covers research articles that showed the discovery of wound healing AMPs from bottle fly larvae which has been used in ancient Maggot debridement therapy for the treatment of chronic infections. This research topic included a wide range of interdisciplinary research that ultimately focused our goals of boosting the knowledge of scientific field of ethnopharmacology for the discovery of lead NAMPs as a novel antibacterial agents.

Cathelicidins are a class of NAMPs that were identified in different animal species, including mamals, birds, fish, reptiles and amphibians (Wang et al., 2008). Several cathelicidin related antimicrobial peptides (CRAMPs) have been discovered from various snake venoms that possesses potent antimicrobial activity. Barros et al. reviewed the current and previous reported data on cathelicidins identified in snake venoms. Authors summarized the data on cathelicidins including its chemistry, characterization, pharmacological action especially antimicrobial and antibioflim effects as well as their mechanism of action. They also highlighted the potentials cathelicidins for the development of novel antibiotics to defend antibiotic-resistant bacteria.

The Solanaceae family consists of approximately 2,700 species that distributed in about 98 genera including both economic and medicinal plants (Olmstead and Bohs, 2007). Plants of the Solanaceae are rich in different bioactive constituents including NAMPs that have been used in different traditional medicinal systems (Chowański et al., 2016). Afroz et al. showed that several bioactive AMPs including defensins, protease inhibitor, lectins, thionin-like peptides, vicilin-like peptides, and snaking were isolated from the plants of Solanaceae family. They highlighted the importance of plants of the Solanaceae as potential sources of NAMPs by summarizing reported AMPs from these plants with their possible molecular mechanism of action as well as further correlated the traditional uses of these plants with their reported AMPs.

Cruciferins, napins, the oil-body proteins and oleosins are the seed storage proteins (SSPs) in mustard and rapeseed (*Brassica napus* L., *B. juncea* L., *B. nigra* L.*, B. rapa* L. and *Sinapis alba* L.), that have been used in traditional medicinal systems against different diseases including infectious diseases (Von Der Haar et al., 2014). Based on the hypothesis that these SSPs might possesses antibacterial and antifungal activities, Rahman et al. investigated both *in silico* and *in vitro* antibacterial activity of napin and cruciferin rapseed proteins to confirm their antimicrobial potential. They found that both the SSPs, napin and cruciferin, can act as a potent candidates to develop new antimicrobial agents as well as can be used in complementary medicine to alleviate diseases.

Small-angle X-ray Scattering (SAXS) is a powerful technique to study protein structures and when the SAXS performed in biological samples it is termed as BioSAXS. BioSAXS helps to understand about the sizes, shape, movement, protein functions in cells which support in drug design and discovery (Chen et al., 2018). Gundlach et al. investigated the effect of two highly active short broad-spectrum AMPs (14D and 69D) against *Escherichia coli* and methicillin-resistant *Staphylococcus aureus* (MRSA) as well as further studied the ultrastructural changes in *E. coli* and MRSA in response to these AMPs using the BioSAXS technique. It was found that the after treatment of either AMP the ultrastructure of *E. coli* and MRSA to be different and the scattering curves for both cells was much more alike. Their results demonstrated that BioSAXS could help to elucidate the mode of action of AMPs that can contribute in the development of antibiotics against resistant bacteria.

Maggot debridement therapy was developed in 1930s to treat chronic ulcers and infections of patients with diabetes and heart diseases (Malekian et al., 2019). Larvae of the green bottle fly *Lucilia sericata* was used for this therapy and the mechanism involved secretion of large amount different AMPs including proline rich peptides (PRPs) into the wound that promote wound healing (van der Plas et al., 2007). To elucidate the mechanism of PRPs, Cytrynska et al. investigated effect of two PRPs (Lser-PRP2 and Lser-PRP3) in *E. coli*. It was found that neither of AMPs interfered with protein synthesis rather bind the bacterial chaperone DnaK that inhibit protein folding. This study indicated that insect AMPs another promising source of antibacterial agents for targeted antimicrobial therapy.

In conclusion, the present research topic includes a wide range of interdisciplinary research work that contributed to our goal of expanding knowledge on NAMPs including their new sources, identification, isolation, characterization, bioactivity, molecular mechanism and the structure-activity features. This research topic successfully gathered comprehensive information on some specific NAMPs related to the field of antibiotic discovery from natural sources. Furthermore, this topic provided insights into potential use of ethnopharmacological approaches to discover new lead NAMPs molecules for antibiotic therapy. We hope that this special collection will help and inspire scientists from different research fields to suitable use of gathered knowledge for the discovery of novel antibiotics using natural AMPs.
